# NaHS Alleviated Cell Apoptosis and Mitochondrial Dysfunction in Remote Lung Tissue after Renal Ischemia and Reperfusion via Nrf2 Activation-Mediated NLRP3 Pathway Inhibition

**DOI:** 10.1155/2021/5598869

**Published:** 2021-04-15

**Authors:** Guangning Zhao, Long Yang, Liming Li, Zhongqiang Fan

**Affiliations:** Department of Urology, Tianjin Medical University General Hospital, Tianjin Heping District Anshan Road 154, Tianjin 300052, China

## Abstract

**Objective:**

Acute kidney injury (AKI) is a common and severe complication in critically ill patients, often caused by renal ischemia-reperfusion (RIR). Previous studies have confirmed that lung injury, rather than renal injury, is one of the leading causes of AKI-induced death. The pathophysiological mechanisms of acute lung injury (ALI) resulting from AKI are very complex and remain unclear. In the present study, we aimed to explore the protective effects and potential mechanism of sodium hydrosulfide (NaHS) on lung injury in RIR mice.

**Methods:**

The RIR model was established in wild-type and Nrf2^−/−^ mice. Different groups of mice were treated with NaHS and MCC950. Lung tissues were harvested to detect lung injury, mitochondrial function, cell apoptosis, the NLRP3 inflammasome, and Nrf2 pathway-related molecules.

**Results:**

RIR led to a deterioration in lung histology, the wet/dry weight ratio, PaO_2_/FiO_2_, and mitochondrial function, in addition to stimulating the activation of the NLRP3 and Nrf2 pathways. MCC950 alleviated mitochondrial dysfunction, lung apoptosis, and histology injury in the lungs after RIR. NaHS treatment markedly improved the lung histological scores, the wet/dry weight ratio, bronchoalveolar lavage fluid (BALF) cell counts, BALF neutrophil counts, BALF neutrophil elastase activity, BALF protein concentration, PaO_2_/FiO_2_, mitochondrial morphology, the red/green fluorescence intensity that indicates changes in mitochondrial membrane potential, respiratory control rate (RCR), ATP, reactive oxygen species (ROS) release, and cell apoptosis via Nrf2-mediated NLRP3 pathway inhibition.

**Conclusion:**

NaHS protected against RIR-induced lung injury, mitochondrial dysfunction, and inflammation, which is associated with Nrf2 activation-mediated NLRP3 pathway inhibition.

## 1. Introduction

Acute kidney injury (AKI) is a common and severe complication in critically ill patients, often caused by renal ischemia-reperfusion (RIR) [1]. During hospitalization, the mortality rate of AKI patients is 5.5-fold higher than that of non-AKI patients [2]. Ischemic AKI rarely occurs in isolation and is normally characterized as part of multiorgan dysfunction. Therefore, the mortality rate of AKI is much higher when combined with extrarenal distal organ injury. Improving renal function by renal replacement therapy alone does not reduce AKI-related mortality, which is mainly due to damage to distal organs. Previous studies have confirmed that lung injury, rather than renal injury, is one of the leading causes of death induced by AKI [3, 4]. Moreover, many clinicians have insisted that patients often die from dysfunction of other organs accompanied by AKI rather than from AKI itself [2]. Until now, there has been no effective medicine or methods for treating AKI.

The pathophysiological mechanisms of acute lung injury (ALI) resulting from AKI are very complex and remain unclear; these mechanisms are mainly characterized by cell apoptosis, pulmonary edema, an acute inflammatory response to the pulmonary parenchyma and alveolar cavity, and impaired alveolar epithelial and pulmonary capillary barrier function, which further leads to inflammatory responses, oxidative stress, and mitochondrial dysfunction [5–7]. In addition, the large capillary network of the lung makes it more sensitive to circulating inflammatory regulators released during RIR and eventually results in ALI [8]. Inflammasome activation is closely related to the inflammatory response. The inflammasome is composed of three functional domains and the adaptor protein apoptosis-associated speck-like protein containing caspase-1 activator domain (ASC), which activates caspase-1 and subsequently promotes maturation of interleukin- (IL-) 1*β* and IL-18 [9]. The NLRP3 inflammasome is regarded as the most characterized inflammasome, which can be triggered by various danger signals. Intriguingly, excessive release of reactive oxygen species (ROS) induced by lung injury has been reported as a vital factor in NLRP3 inflammasome activation. In addition, nuclear factor E2-related factor 2 (Nrf2) is considered an important nuclear transcription factor that is extensively involved in inhibiting oxidative stress and subsequent inflammation [10].

Hydrogen sulfide (H_2_S), an important gas signaling molecule, is involved in a series of physiological processes, such as inflammatory disease pathogenesis and associated organ injury; these injuries include sepsis [11], ischemia/reperfusion injury [12], kidney injury [13], and lung injury [14]. It is widely accepted that normal levels of circulating H_2_S are vital to human health. However, excessive H_2_S can damage oxidative phosphorylation of the respiratory chain and further play a role in energy metabolism, causing cell death via binding to mitochondrial cytochrome oxidase C [15]. Interestingly, sufficient exogenous H_2_S exerts protective effects on organs in different disease models. It has been reported that H_2_S pretreatment prevented neutrophil transmigration to ameliorate lipopolysaccharide- (LPS-) induced ALI by inhibiting proinflammatory cytokines and oxidative signaling in neutrophils [16]. Furthermore, exogenous H_2_S administration alleviated liver injury induced by paraquat (PQ) by promoting antioxidative capability, inhibiting the activation of the ROS-induced NLRP3 inflammasome, and improving mitochondrial function in an Nrf2-dependent manner [17]. Nevertheless, the effect of H_2_S on RIR-induced acute lung injury remains unclear. It is well recognized that H_2_S is noxious, toxic, and uncontrollable; for this reason, NaHS was used as an H_2_S donor in our study. In the present study, we mainly investigated whether NaHS treatment has a protective effect against RIR-induced ALI. Furthermore, we explored the potential molecular mechanisms by which the Nrf2 and NLRP3 pathways are involved in organ injury, inflammatory response, cell apoptosis, and mitochondrial dysfunction in the lung tissue after RIR.

## 2. Materials and Methods

### 2.1. Ethics Statement and Animal Preparation

We purchased wild-type (WT) and Nrf2^−/−^ C57BL/6J mice (both 8 weeks old and 18–25 g in weight) from the Laboratory Animal Center of the Military Medical Science Academy of the Chinese People's Liberation Army (PLA). All mice were caged under a regular 12 h/12 h light/dark cycle and fed with food and water. This experimental protocol was authorized by the Institutional Animal Care and Use Committee of Tianjin Medical University and performed according to the guidelines of the National Institutes of Health for the Care and Use of Experimental Animals. Anesthesia was induced by intraperitoneal injection of pentobarbital sodium (40 mg/kg in normal saline), and euthanasia was performed by CO_2_ inhalation under deep anesthesia.

### 2.2. RIR Injury and Treatment

All mice were anesthetized with sevoflurane. During the experiment, mice were placed in an infant incubator at 32°C with warm saline. Bilateral lateral incisions were performed to reveal the kidney. The right kidney was removed, and the left renal arteriovein was occluded with a small vessel clip for 30 min [18]. Reperfusion was initiated by the removal of the clamp. Subsequently, the abdominal muscles and skin were sutured separately. In the sham group, the left renal arteriovein was not blocked and reperfusion was not performed. Treatment groups were intraperitoneally (i.p.) injected with NaSH (50 *μ*mol/kg body weight (BW)) and/or MCC950 (4 mg/kg BW). Lung tissues were taken 24 h later for follow-up experiments.

### 2.3. Western Immunoblot Analysis

Mice were deeply anesthetized before decapitation, lung tissues were rapidly removed after 24 h of RIR, and tissues were homogenized after freezing. Protein extracts were obtained by grinding tissues in RIPA buffer containing protease inhibitors. Proteins were loaded in a polyacrylamide SDS gel, electrophoresis was performed, and separated proteins were transferred to a PVDF membrane. The membrane was blocked in 5% bovine serum albumin (BSA) and incubated with appropriate primary antibodies overnight at 4°C. The primary antibodies contained anti-NLRP3 (Abcam, ab263899, 1 : 1000 dilution), anti-caspase-1 P10 (Abcam, ab179515, 1 : 1000 dilution), anti-IL-1*β*P17 (Abcam, ab234437, 1 : 1000 dilution), anti-Nrf2 (Abcam, ab89443, 1 : 1000 dilution), anti-histone (Abcam, ab1791, 1 : 5000 dilution), anti-HO-1 (Abcam, ab68477, 1 : 10000 dilution), anti-NQO1 (Abcam, ab28947, 1 : 1000 dilution), anti-Trx (Abcam, ab273877, 1 : 1000 dilution), anti-Bax (Abcam, ab32503, 1 : 10000 dilution), anti-Bcl-2 (Abcam, ab182858, 1 : 2000 dilution), and anti-GAPDH antibody (Abcam, ab8245, 1 : 10000 dilution). The membranes were incubated for 1 h at room temperature with the corresponding secondary antibodies, followed by washing five times with Tris-buffered saline Tween-20. The bands were treated with enhanced chemiluminescence (ECL) reagent and then exposed to ECL-Hyperfilm (Amersham Biosciences, Piscataway, NJ, USA).

### 2.4. RNA Extraction and Reverse Transcription Polymerase Chain Reaction (RT-PCR)

The RNA of cells was extracted using an RNA Extraction Kit (Invitrogen). The RNA concentration was quantified using a spectrophotometer (Nanodrop 2000; Thermo Fisher Scientific). Complementary DNA synthesis was performed using the Prime Script RT reagent kit (Takara, Osaka, Japan). Quantitative RT-PCR was performed using gene-specific primers and SYBR Green PCR Master Mix (Takara) on a Roche light cycler 480 real-time PCR system as follows: 95°C for 30 s, followed by 40 cycles of 95°C for 15 s and then 60°C for 30 min. Measurements were performed in triplicate and normalized to endogenous GAPDH levels. Relative fold change was calculated using the 2-*ΔΔ*Ct method. The sequences of primers for IL-1*β*, NLRP3, Nrf2 caspase-1, and GAPDH were as follows: IL-1*β*, sense CTGAGCTCGCCAGTGAAATG, antisense TGTCCATGGCCACAACAACT; NLRP3, sense AAGGGCCATGGACTATTTCC, antisense GACTCCACCCGATGACAGTT; Nrf2, sense GAGAGCCCAGTCTTCATTGC, antisense TTGGCTTCTGGACTTGGAAC; caspase-1, sense CTCAGGCTCAGAAGGGAATG, antisense CGCTGTACCCCAGATTTTGT; and GAPDH, sense AAGAAGGTGGTGAAGCAGGC, antisense TCCACCACCCAGTTGCTGTA.

### 2.5. Transmission Electron Microscopy

We resected specimens, fixed them in 0.1 M phosphate buffer solution with fixation buffer containing 2% paraformaldehyde and 2.5% glutaraldehyde, and stored them at 4°C until embedding. We then postfixed tissue specimens in 1% phosphate-buffered osmium tetroxide and embedded them in Spurr's resin. Tissues were sectioned at 0.12 *μ*m and stained with 0.2% lead citrate and 1% uranyl acetate. Images were examined with a Jeol TEM-2000EX II microscope (Jeol Ltd., Tokyo, Japan).

### 2.6. Matrix Metalloproteinase (MMP) Assay

We resected specimens, fixed them in 0.1 M phosphate-buffered solution with fixation buffer with 2% paraformaldehyde and 2.5% glutaraldehyde, and kept them at 4°C. Then, the fixed tissue specimens were placed in 1% phosphate-buffered osmium tetroxide and embedded in Spurr's resin. Specimens were sectioned at 0.12 *μ*m and stained with 0.2% lead citrate and 1% uranyl acetate. Images were examined with a Jeol TEM-2000EX II microscope (Jeol Ltd.).

### 2.7. Isolated Mitochondrial Respiratory Test

The index was measured by measuring the oxygen consumption rate in a 30°C water bath using a Clark electrode with a 500 *μ*L reaction chamber [19]. The chamber was filled with fresh breathing buffer until a stable baseline was tested. Freshly isolated mitochondria (500 *μ*g) were placed into the chamber, followed by a waiting period until the oxygen consumption rate stabilized. Upon addition of succinate (5 mm), a low oxygen consumption rate (State IV) was tested. ADP (5 mm) was then added to stimulate a high-oxygen consumption rate (state III), which was switched back to the IV rate due to the consumption of ADP. The respiratory control rate (RCR) (State III rate/State IV rate) is complicated and may indicate the capacity of mitochondrial oxidative phosphorylation.

### 2.8. ATP and ROS Determination

ATP content was measured using an ATP bioluminescence assay kit (Roche Diagnostics, Laval, Quebec, Canada) using a TD-20/20 luminometer (Turner Designs, Sunnyvale, CA, USA) following the manufacturer's instructions with an integration time of 10 s. We determined ROS levels using a kit according to the manufacturer's instructions (CA1410, Solarbio Scientific Technology). After the procedure, 500 *μ*L of 10 *μ*m DCFH-DA (dissolved in serum-free medium) was added into each well and incubated at 37°C for 20 min in the dark. The loading buffer was replaced with serum-free medium and washed three times to remove residual DCFH-DA. The cells were then observed using an inverted immunofluorescence microscope (TH4-200; Olympus, Tokyo, Japan) equipped with an argon laser (488 nm). The public domain program ImageJ v. 1.47 (National Institutes of Health, Bethesda, MD, USA) was used to collect and process the image at the single-cell level on the Windows operating system for fluorescence measurement.

### 2.9. TUNEL Staining

Mice were deeply anesthetized 24 h after reperfusion. The lungs were quickly removed and cut into slices using a microtome (Leica CM 1850 Cryostat, Nussloch, Germany). TUNEL staining was performed using an In Situ Cell Death Detecting Kit, Fluorescein (Roche Applied Science, Mannheim, Germany) following the corresponding protocol. In brief, specimens were fixed in 4% paraformaldehyde for 30 min, permeabilized with 0.01% Triton X-100 (Sigma) in 0.1% sodium citrate for 2 min on ice, and then incubated for 60 min at 37°C with the TUNEL reaction mixture in a humidified atmosphere. Every fifth section of each slice was collected, and the TUNEL-positive cells were counted under a fluorescence microscope (NIKON ECLIPSE-80i, Nikon, Japan) at 400x magnification.

### 2.10. Pathological Studies

We followed the methods of Li et al. [20]. The right lower lobe of the lung was fixed with 10% neutral-buffered formalin for 24 h, embedded in paraffin, stained with hematoxylin and eosin (H&E), and observed under a light microscope. A semiquantitative scoring system was used to evaluate alveolar congestion, alveolar hemorrhage, neutrophil infiltration or aggregation in the alveolar cavity or vascular wall, alveolar wall/hyaline membrane thickness, and inflammatory cell infiltration. The scoring criteria for the pathological results were as follows: 0 = no injury, 1 = slight injury (25%), 2 = moderate injury (50%), 3 = severe injury (75%), and 4 = very severe injury (almost 100%). As mentioned earlier, the results for each project range from 0 to 4 [21]. The sum of the four variables represents the lung injury score (total score: 0–16).

### 2.11. Immunohistochemical Staining

We followed the methods of Li et al. [20]. After anesthesia, the mice were injected with isotonic sodium chloride solution and 4% paraformaldehyde. The lung was fixed with 4% paraformaldehyde for 6 h. The lung tissue was embedded in paraffin and sectioned at 5 *μ*m for immunohistochemical staining. After dewaxing, hydration, and antigen recovery, the sections were blocked in 5% goat serum and then with anti-Nrf2 rabbit monoclonal antibody (1 : 500) overnight at 4°C. After washing with phosphate-buffered saline (PBS) three times, the sections were incubated with secondary antibody (1 : 200) at 37°C for 1 h and stained with diaminobenzidine. Hematoxylin staining was performed for 5 min. The intracellular localization of Nrf2 was then observed using an Olympus eclipse 80i microscope (Olympus), and the images were analyzed with Image-Pro Plus 6.1 System. All slides were analyzed by a blinded method.

### 2.12. Lung Wet-to-Dry (*W*/*D*) Weight Ratios

The *W*/*D* weight ratio of the lung was calculated to evaluate the severity of pulmonary edema. The harvested left lobe was washed with normal sodium to remove excess water. The lung tissue was then weighed as wet weight. The sample was dried to a constant weight at 70°C in an electric air dryer for 24 h, and then the dry weight was recorded. The *W*/*D* weight ratio was then determined.

### 2.13. Oxygenation Index Analysis

To determine the oxygenation capability of the lung, the ratio of oxygen tension to the fraction of inhaled oxygen (PaO_2_/FiO_2_) was measured. At 24 h of renal ischemia reperfusion, animals were anesthetized and intubated with a 20-gauge catheter. They were mechanically ventilated with 7 mL/kg pure oxygen. The respiratory rate was 120 breaths/min. The animals were ventilated for 15 min before blood gas sampling. Arterial blood was obtained from the carotid artery and measured with a GEM Premier 3000 gas analyzer (Instrumentation Laboratory, Milan, Italy).

### 2.14. Cell Counts and Protein Concentration in Bronchoalveolar Lavage Fluid (BALF)

BALF was collected by bronchoalveolar lavage using the above method [22]. BALF was obtained by tracheal intubation with a 20-gauge catheter. A double volume of 0.5 mL PBS (pH 7.4) was instilled, gently aspirated, combined, and reaspirated. The lavage samples were centrifuged at 4°C at 1,500 × *g* for 10 min. The supernatant was stored at -20°C. In addition, the cell precipitates were resuspended in PBS, and the total cell count was measured using a blood cell counter (Beckman Coulter, Inc., Fullerton, CA, USA). The slides were visualized by Wright-Giemsa staining (Fisher Scientific Co., Middletown, VA, USA), and polymorphonuclear leukocytes (PMNs) were identified by certified laboratory technicians using a blinded method. Total protein concentration in the BALF was determined using standard commercial kits (Bio-Rad Laboratories, Hercules, CA, USA). The concentration of neutrophil elastase was determined by ELISA (BioVendor, Brno, Czech Republic).

### 2.15. Statistical Analysis

All data are shown as the mean ± standard deviation (SD) from the independent experiments. An unpaired *t*-test (if the values had a Gaussian distribution) or the Mann–Whitney *U*-test (if the values did not have such a distribution) was used to analyze the differences between the two groups, and one-way analysis of variance (ANOVA) with a Tukey post hoc test was used to analyze the differences among groups (three or more groups). *P* < 0.05 was considered statistically significant, and the significance test was two-tailed. GraphPad Prism software (version 7.0; San Diego, CA, USA) and SPSS statistical software (version 20.0; IBM, Inc., Chicago, Illinois, USA) were used for statistical analysis.

## 3. Results

### 3.1. NaHS Attenuated RIR-Induced Lung Injury and NLRP3 Pathway Activation in Mice

**Figure 1:**
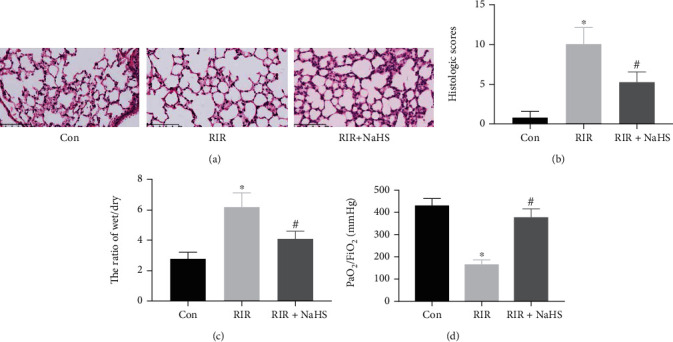
The effect of sodium hydrosulfide hydrate (NaHS) on lung histological injury, the wet/dry weight ratio, and the oxygenation index (PaO_2_/FiO_2_). Lung tissues were stained with H&E ((a), ×400), the scores of H&E staining in the lung tissue were counted (b), and the wet/dry weight ratio (c), and PaO_2_/FiO_2_ ratio of lung tissues (d) are shown as a bar graph. Values are presented as the mean ± SD. ^∗^*P* < 0.05 vs. Con; ^#^*P* < 0.05 vs. RIR; *n* = 6.

In the present study, we first investigated the effect of NaHS on lung injury after RIR. Histological changes were assessed by H&E staining, and the *W*/*D* weight ratio and oxygenation index were detected in the lung tissue at 24 h after RIR. The results of H&E staining showed that compared with the control (Con) group, RIR led to severe lung injury with almost complete destruction of the pulmonary architecture, alveolar space disappearance, infiltration of neutrophils into lung interstitium and alveolar space, and interstitial edema. NaHS administration improved lung injury, with the improvement characterized by intact alveolar architecture, reduced interstitial edema, and decreased alveolar infiltration of inflammatory cells (Figure 1(a)). Furthermore, a scoring system was used to grade the degree of lung injury. RIR induced a significant upregulation in lung histological scores when compared with the Con group, which was reduced by NaHS administration (Figure 1(b), *P* < 0.05). Meanwhile, when compared with the Con group, the *W*/*D* weight ratio of lung tissue was increased by RIR challenge, while NaHS administration significantly decreased this ratio (Figure 1(c), *P* < 0.05). Interestingly, the PaO_2_/FiO_2_ ratio was deteriorated by RIR when compared with the Con group, which showed improvement upon NaHS administration (Figure 1(d), *P* < 0.05).

**Figure 2:**
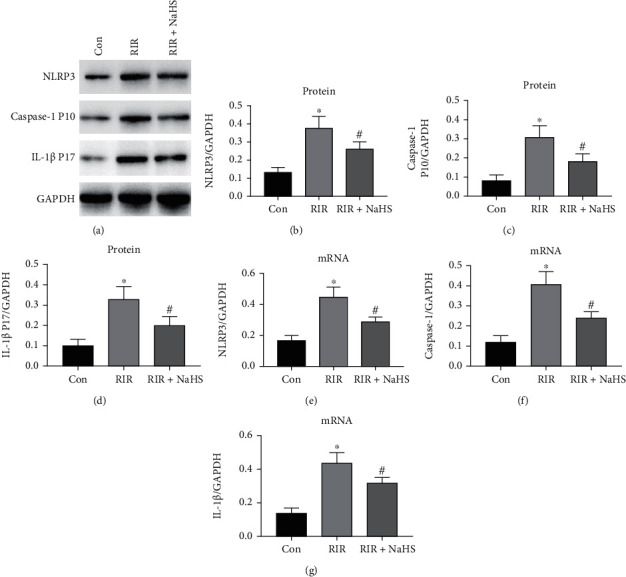
NaHS attenuated NLR family pyrin domain containing 3 (NLRP3), caspase-1, and interleukin 1 beta (IL-1*β*) protein and mRNA expression in the lung tissue after renal ischemia reperfusion (RIR). The expression levels of NLRP3, caspase-1, and IL-1*β* were measured by western blot (a). The quantifications of NLRP3 protein expression (b), caspase-1 protein expression (c), and IL-1*β* protein expression (d) are shown as bar graphs. The quantifications of NLRP3 mRNA expression (e), caspase-1 mRNA expression (f), and IL-1*β* mRNA expression (g) are shown as bar graphs, as measured by RT-PCR assay. Values are presented as the mean ± SD. ^∗^*P* < 0.05 vs. Con; ^#^*P* < 0.05 vs. RIR; *n* = 3.

The NLRP3 inflammasome is reported to be closely associated with the pathophysiological process of lung injury. As shown in Figure 2, RIR upregulated the protein and mRNA expression of NLRP3 and promoted the cleavage of caspase-1 compared with the Con group. This subsequently promoted IL-1*β* in the lung tissue, and these effects were significantly attenuated by NaHS administration (Figures 2(a)–2(g), *P* < 0.05). These results indicated that NaHS administration exhibited a significant protective effect against RIR-induced histopathological injury and dysfunction in the lung tissue. Moreover, the protective effect of NaHS was blocked by activation of the NLRP3 pathway.

### 3.2. NLRP3 Inhibition by MCC950 Alleviated Mitochondrial Dysfunction, Lung Cell Apoptosis, and Histology Injury in the Lung after RIR

**Figure 3:**
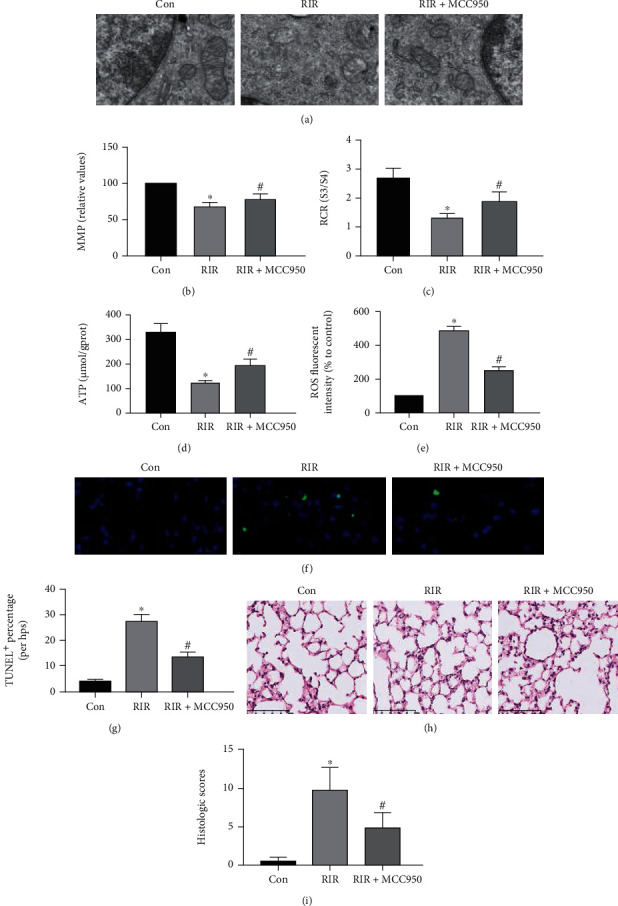
The effect of MCC950 on mitochondrial morphology, matrix metalloproteinase (MMP), respiratory control rate (RCR), adenosine-triphosphate (ATP), reactive oxygen species (ROS), apoptosis, and lung injury after renal ischemia reperfusion (RIR). The mitochondrial morphology was observed by TEM ((a), *n* = 3). Bar graphs show the red/green fluorescence intensity (b), RCR (c), ATP (d), and ROS (e). The fluorescence image (f) and percentage of TUNEL-positive apoptotic cells (g) were measured by TUNEL assay. Lung injury was observed by H&E (h, ×400), and the H&E staining scores in the lung tissue were counted (i). Values are presented as the mean ± SD. ^∗^*P* < 0.05 vs. Con; ^#^*P* < 0.05 vs. RIR; *n* = 6 except (a).

To further confirm the role of NLRP3 in lung injury, we used MCC950 to inhibit NLRP3 expression. From the present results, we found that RIR caused abnormalities in mitochondrial morphology, characterized by mitochondrial swelling, disorganized and fragmented cristae, and mitochondrial dysfunction. These findings showed that MMP, RCR, ATP, and ROS release were deteriorated in the lung tissue in the RIR group compared with in the Con group (Figures 3(a)–3(e), *P* < 0.05). The inhibition of NLRP3 by MCC950 obviously improved mitochondrial morphology and dysfunction by ameliorating mitochondrial morphology abnormalities; increasing MMP, RCR, and ATP levels; and decreasing the release of ROS (Figures 3(a)–3(e), *P* < 0.05). TUNEL assay indicated that RIR resulted in the upregulation of cell apoptosis, and H&E demonstrated that histological injury was upregulated in the lung tissue; these effects were alleviated by the inhibition of NLRP3 via MCC950 administration (Figures 3(f)–3(i), *P* < 0.05). Although this inhibition was incomplete, it was still statistically significant. Therefore, these data suggest that inhibition of NLRP3 could alleviate mitochondrial morphological abnormalities and mitochondrial dysfunction, cell apoptosis, and tissue histology injury in the lungs after RIR.

### 3.3. NaHS Regulated NLRP3 and Its Downstream Signaling Molecules via the Nrf2 Pathway in RIR-Induced Lung Injury

**Figure 4:**
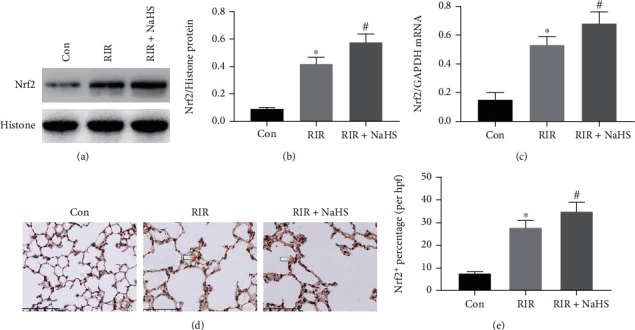
The effect of sodium hydrosulfide hydrate (NaHS) on NF-E2-related factor 2 (Nrf2) protein and mRNA expression in the lung tissue after renal ischemia reperfusion (RIR). The expression levels of Nrf2 were measured by western blot (a). The quantification of Nrf2 (b) is shown as a bar graph. The quantification of Nrf2 mRNA expression (c) is shown as a bar graph, which was measured by RT-PCR assay. The Nrf2-positive cells were stained by immunochemical staining (d). The percentage of Nrf2-positive cells is shown as a bar graph (e). Values are presented as the mean ± SD. ^∗^*P* < 0.05 vs. Con; ^#^*P* < 0.05 vs. RIR; *n* = 3.

To investigate the effect of NaHS on Nrf2 expression, we measured the mRNA and protein expression of Nrf2 in the lung tissue after RIR. As shown in Figure 4, Nrf2 protein expression in the nucleus and mRNA expression were increased compared with that in the Con group (Figures 4(a)–4(c), *P* < 0.05). We also observed the percentage of Nrf2-positive cells in the lung tissue by immunohistochemical assays. The results showed that the percentage of Nrf2-positive cells showed the same increasing trend as Nrf2 protein and mRNA (Figures 4(d) and 4(e), *P* < 0.05). As detected by western blotting, RT-PCR, and immunohistochemical assays, Nrf2 expression was further activated and upregulated after NaHS treatment in the RIR+NaHS group when compared with the RIR group (Figures 4(a)–4(e), *P* < 0.05). These results indicated that RIR stimulated Nrf2 activation and upregulation in the lung tissue, which was further increased by NaHS treatment.

**Figure 5:**
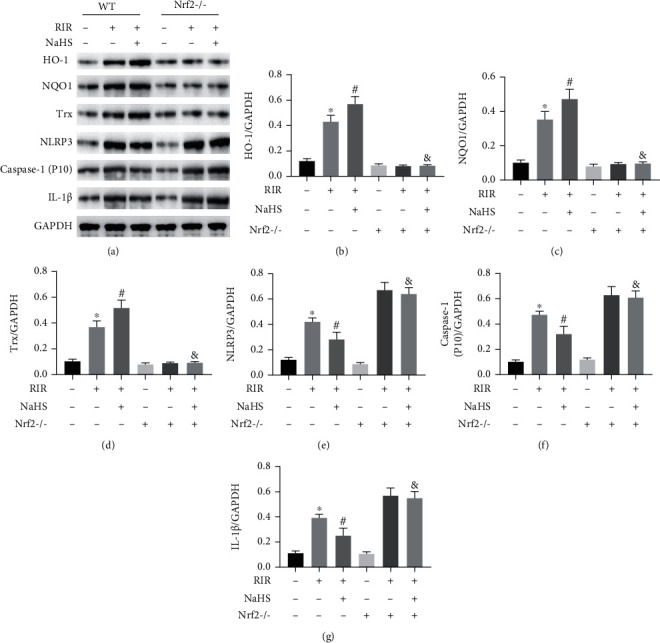
Sodium hydrosulfide hydrate (NaHS) regulated heme oxygenase-1 (HO-1), NADPH quinone dehydrogenase 1 (NQO1), thioredoxin (Trx), NLR family pyrin domain containing 3 (NLRP3), caspase-1, and interleukin 1 beta (IL-1*β*) in the lung tissue after renal ischemia reperfusion (RIR) in WT and Nrf2^−/−^ mice. The expression levels of HO-1, NQO1, Trx, NLPR3, caspase-1, and IL-1*β* were measured by western blot (a). The quantifications of HO-1 (b), NQO1 (c), Trx (d), NLPR3 (e), caspase-1 (f), and IL-1*β* (g) are shown as bar graphs. Values are presented as the mean ± SD. ^∗^*P* < 0.05 vs. the RIR-NaHS-Nrf2^−/−^ group; ^#^*P* < 0.05 vs. the RIR+NaHS-Nrf2^−/−^ group; ^&^*P* < 0.05 vs. the RIR+NaHS+Nrf2^−/−^ group; *n* = 3.

Nrf2^−/−^ mice were used to further determine the effect of Nrf2 on its downstream molecules and the NLRP3 pathway after NaHS treatment (Figure 5). RIR led to increased expression of the Nrf2 downstream molecules HO-1, NQO1, and Trx in the control group compared to that in the RIR group, and the amount of NLPR3, cleavage of the adaptor caspase-1, and mature IL-1*β* also increased. Meanwhile, NaHS treatment markedly increased HO-1, NQO1, and Trx expression and reduced NLPR3, caspase-1, and IL-1*β* expression in the RIR+NaHS group compared to that in the RIR group of WT mice (Figures 5(a)–5(g), *P* < 0.05). NaHS further improved HO-1, NQO1, and Trx expression in the lung tissue of the RIR group of WT mice. Nrf2 knockout abolished the regulatory effect of NaHS on HO-1, NQO1, and Trx in the RIR+NaHS group of Nrf2^−/−^ mice (Figures 5(a)–5(d), *P* < 0.05). Moreover, NaHS alleviated the RIR-induced expression of NLPR3, caspase-1, and IL-1*β* in the lung tissue of WT mice, but not in Nrf2^−/−^ mice. These results indicate that NaHS regulates NLRP3 and its downstream signaling molecules via the Nrf2 pathway in the lung tissue of mice with RIR.

### 3.4. NaHS Improved the Mitochondrial Morphology and Mitochondrial Dysfunction in the Lung Tissue of RIR Mice via Nrf2-Mediated NLRP3 Pathway Inhibition

**Figure 6:**
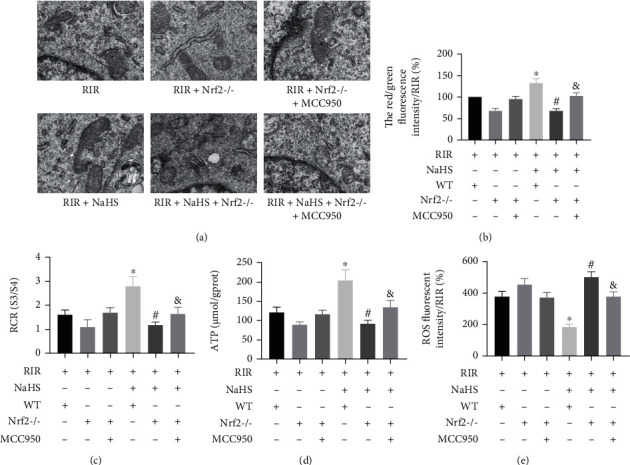
Sodium hydrosulfide hydrate (NaHS) regulated mitochondrial morphology, matrix metalloproteinase (MMP), respiratory control rate (RCR), adenosine-triphosphate (ATP), and reactive oxygen species (ROS) in the lung tissue after RIR in wild-type (WT) and NF-E2-related factor 2 knockout (Nrf2^−/−^) mice. The mitochondrial morphology was observed by TEM ((a), *n* = 3). Bar graphs show the red/green fluorescence intensity (b), RCR (c), ATP (d), and ROS (e). Values are presented as the mean ± SD. ^∗^*P* < 0.05 vs. the RIR+NaHS-WT+ group; ^#^*P* < 0.05 vs. the RIR+NaHS+WT+ group; ^&^*P* < 0.05 vs. the RIR+NaHS+Nrf2^−/−^ group; *n* = 6 except (a).

Compared with the RIR group of WT mice, NaHS obviously improved mitochondrial morphology and mitochondrial dysfunction, showing that the red/green fluorescence intensity (detecting changes in mitochondrial membrane potential), RCR, ATP, and ROS release were improved in the lung tissue (Figures 6(a)–6(e), *P* < 0.05). Abnormalities in the mitochondrial morphology and mitochondrial dysfunction were decreased in the RIR+NaHS group of Nrf2^−/−^ mice compared to WT mice, suggesting that NaHS did not improve morphology abnormality, nor did it regulate the red/green fluorescence intensity, RCR, ATP, and ROS release in the absence of Nrf2 (Figures 6(a)–6(e), *P* < 0.05). Compared with the RIR+NaHS group of Nrf2^−/−^ mice, MCC950 treatment partly reversed the effect of the absence of Nrf2 on mitochondrial morphology abnormality and mitochondrial dysfunction in the RIR+NaHS+MCC950 group of Nrf2^−/−^ mice (Figures 6(a)–6(e), *P* < 0.05). These data suggested that NaHS exerted a protective effect on mitochondrial morphology and dysfunction in the lung tissue of RIR mice via Nrf2-mediated NLRP3 pathway inhibition.

### 3.5. NaHS Decreased Cell Apoptosis in the Lung Tissue of RIR Mice via Nrf2-Mediated NLRP3 Pathway Inhibition

**Figure 7:**
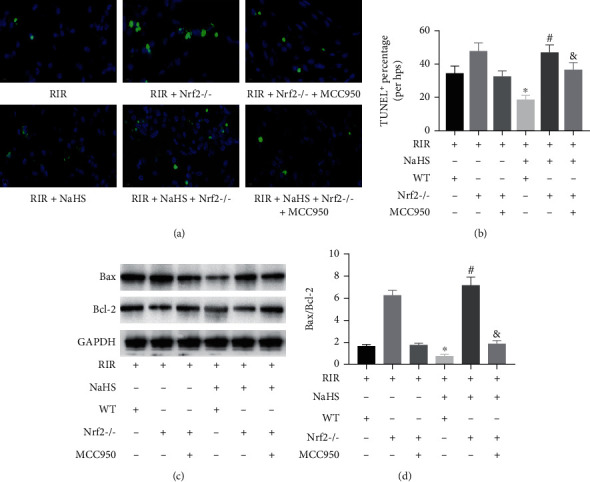
Sodium hydrosulfide hydrate (NaHS) alleviated cell apoptosis in the lung tissue after renal ischemia reperfusion (RIR) in wild-type (WT) and NF-E2-related factor 2 knockout (Nrf2^−/−^) mice. Fluorescence images were taken (a), and the percentage of TUNEL-positive apoptotic cells (b) was measured by TUNEL assay. The lung tissue was stained via H&E ((h), ×400). The expression levels of Bax and Bcl-2 were measured by western blot (c). The quantifications of Bax and Bcl-2 are shown as bar graphs (d). Values are presented as the mean ± SD. ^∗^*P* < 0.05 vs. the RIR+NaHS-WT+ group; ^#^*P* < 0.05 vs. the RIR+NaHS+WT+ group; ^&^*P* < 0.05 vs. the IR+NaHS+Nrf2^−/−^ group; *n* = 3.

As shown in Figure 7, NaHS treatment obviously reduced the percentage of TUNEL-positive cells, Bax expression, and Bax/Bcl-2 ratio and increased Bcl-2 expression in the lung tissue with RIR compared to the RIR group of WT mice (Figures 7(a)–7(d), *P* < 0.05). When compared with the RIR+NaHS group of WT mice, the TUNEL-positive cell percentage, Bax expression, and Bax/Bcl-2 were elevated and Bcl-2 expression was reduced in the RIR+NaHS group of Nrf2^−/−^ mice (Figures 7(a)–7(d), *P* < 0.05). However, MCC950 treatment significantly reduced the TUNEL-positive cell percentage, Bax expression, and Bax/Bcl-2 expression and increased Bcl-2 expression in the RIR+NaHS+MCC950 group of Nrf2^−/−^ mice compared to those in the RIR+NaHS group of Nrf2^−/−^ mice (Figures 7(a)–7(d), *P* < 0.05). These results indicated that NaHS attenuated cell apoptosis in the lung tissue of RIR mice via Nrf2-mediated NLRP3 pathway inhibition.

### 3.6. NaHS Improved Lung Injury in the Lung Tissue of RIR Mice via Nrf2-Mediated NLRP3 Pathway Inhibition

**Figure 8:**
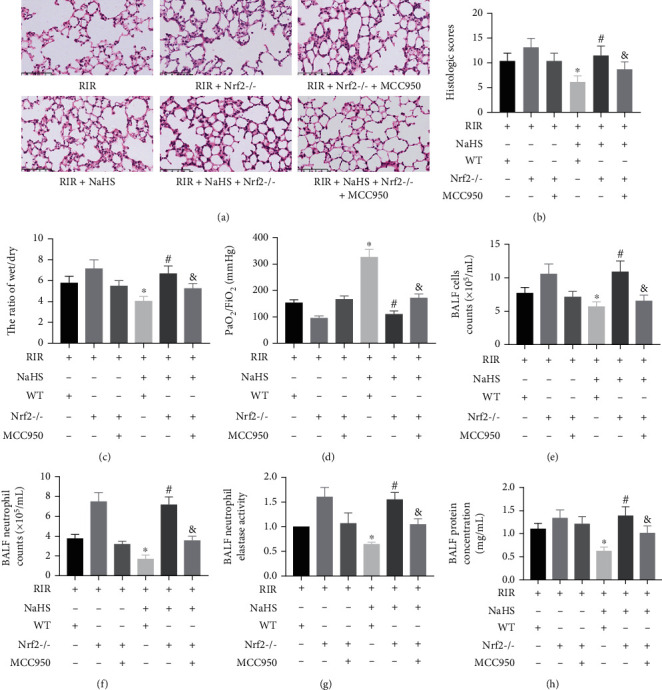
Sodium hydrosulfide hydrate (NaHS) improved lung injury, the wet/dry weight ratio, PaO_2_/FiO_2_ ratio, cell counts, neutrophil counts, neutrophil elastase activity, and protein concentration of bronchoalveolar lavage fluid (BALF) in the lung tissue after renal ischemia reperfusion (RIR) in wild-type (WT) and NF-E2-related factor 2 knockout (Nrf2^−/−^) mice. Lung tissues were stained with H&E ((a), ×400), and the H&E staining scores in lung tissue were counted (b). The wet/dry weight ratio (c) and PaO_2_/FiO_2_ ratio of lung tissue (d) are shown as a bar graph. Cell counts (e), neutrophil counts (f), neutrophil elastase activity (g), and protein concentration (h) of BALF are shown as a bar graph. Values are presented as the mean ± SD. ^∗^*P* < 0.05 vs. the RIR+NaHS-WT+ group; ^#^*P* < 0.05 vs. the RIR+NaHS+WT+ group; ^&^*P* < 0.05 vs. the IR+NaHS+Nrf2^−/−^ group; *n* = 6.

In WT mice, NaHS administration markedly alleviated lung injury and improved the histological scores, W/D weight ratio, BALF cell counts, BALF neutrophil counts, BALF neutrophil elastase activity, BALF protein concentration, and the PaO_2_/FiO_2_ ratio in the RIR+NaHS group compared to those in the RIR group (Figures 8(a)–8(h), *P* < 0.05). The absence of Nrf2 obviously abolished the protective effect of NaHS on lung injury and histological scores, the W/D weight ratio, BALF cell counts, BALF neutrophil counts, BALF neutrophil elastase activity, BALF protein concentration, and the PaO_2_/FiO_2_ ratio in the RIR+NaHS group of Nrf2^−/−^ mice compared to WT mice. Nevertheless, in the RIR+NaHS+MCC950 group of Nrf2^−/−^ mice, the inhibition of NLRP3 by MCC950 treatment partly reversed the effect of the absence of Nrf2 on the above indicators compared with the RIR+NaHS group. These results indicated that NaHS improved lung injury in RIR mice via Nrf2-mediated NLRP3 pathway inhibition.

## 4. Discussion

Ischemia-reperfusion injury is a common perioperative complication. Ischemia-reperfusion injury after renal transplantation often causes acute renal failure and acute kidney injury, which may be associated with extrarenal organ dysfunction and pathological injury. Pulmonary capillaries are abundant and extremely sensitive to inflammatory factors in the RIR injury response, and acute lung injury is easily induced once RIR injury occurs [2]. The inflammatory response is one of the most important characteristics of ischemia-reperfusion injury. After RIR, a large number of proinflammatory cytokines and chemokines are produced, including IL-1*β*, IL-6, and TNF-*α*. The release of cytokines into the bloodstream can exacerbate the stress response and interfere with immune system function, leading to lung damage. Our results support the idea that RIR induced lung injury, lung edema, a decline in the oxygenation index (PaO_2_/FiO_2_), the upregulation of BALF cell counts, BALF neutrophil counts, BALF neutrophil elastase activity, and BALF protein concentration and increased the number of TUNEL-positive cells. These data suggest that RIR induced remote lung injury, lung inflammation infiltration, and cell apoptosis.

As a key gas signaling molecule, H_2_S plays a complex role in a wide spectrum of phenomena in different systems and organs, including inflammation, apoptosis, and oxidative stress. Notably, H_2_S participates in both physiological and pathological processes in various kidney diseases [23, 24]. However, the therapeutic effect of exogenous H_2_S on remote organ injury and the related mechanisms remain to be elucidated. We therefore designed this study to investigate the effect of exogenous H_2_S (NaHS) on RIR-induced lung injury and to further explore the underlying mechanism. More intriguingly, we found that NaHS could attenuate lung histological injury scores and the W/D weight ratio and increase the oxygenation index (PaO_2_/FiO_2_) induced by RIR.

Inflammasomes are high molecular weight protein complexes synthesized intracellularly by pattern recognition receptors (PRRs). The NLRP3 inflammasome, which is mainly composed of NLRP3 protein, pro-caspase-1, and ASC, has been recognized as a dynamic stabilization sensor for a wide range of cells. Once activated, it directly mediates the recruitment of procaspase-1 and activates caspase-1, which in turn controls cytosolic precursor factors including pro-IL-1*β* and pro-IL-18 by cleavage. This cleavage allows these factors to mature into cytokines such as IL-1*β* and IL-18. The NLRP3 inflammasome activates pathways and their associated products and plays an important role in the development of various inflammatory responses and metabolic and autoimmune diseases. NLRP3 inflammasomes respond to various risk states of the body (including infection and metabolic disorders) after inflammatory factors (IL-1*β*, IL-18) are released, mainly causing lung injury [25]. IL-1*β* upregulates the expression of adhesion factors in pulmonary capillary endothelial cells, which in turn promotes the chemotaxis and activation of inflammatory cells into the lung tissue. This process releases a large number of inflammatory factors and triggers the inflammatory “waterfall” cascade amplification effect; it can also destroy the cell surface localization of vascular endothelial cell cadherin by promoting endocytosis, increasing capillary permeability, and promoting pulmonary edema [26]. In our study, we found that NLRP3, caspase-1, and IL-1*β* are activated and upregulated in lung tissue injury induced by RIR challenge. Inhibition of the NLRP3 inflammasome by MCC950 reversed mitochondrial dysfunction, lung injury, and apoptosis in RIR-induced lung injury. Moreover, abnormalities in mitochondrial morphology, mitochondrial dysfunction, cell apoptosis, and histology injury scores were improved. More interestingly, NaHS had the same effect as MCC950 on the NLRP3 inflammasome pathway, showing that NLRP3, caspase-1, and IL-1*β* were inhibited by NaHS treatment.

Nrf2 is a transcription factor that can effectively scavenge ROS, is widely present in human tissues, and is involved in the regulation of physiological processes in the body [27]. Nrf2 is a key regulator of endogenous antioxidative stress. When cells sense oxidative stress, Nrf2 can translocate from the cytoplasm to the nucleus and promote the transcription of antioxidant enzymes, followed by the clearance of excessive ROS and metabolites with cytotoxic effects in cells, thereby maintaining the homeostasis of the intracellular environment. Recently, an increasing number of studies have found that Nrf2 plays a key role in regulating the NLRP3 pathway [28]. ROS-induced oxidative stress initiates NLRP3 inflammasome activation, while the absence of Nrf2 affects the expression of NLRP3 and its downstream signaling molecules. Nevertheless, NLRP3 silencing did not affect the expression of the Nrf2 pathway, indicating that Nrf2/HO-1 signaling could be a key pathway for activating the NLRP3 inflammasome [29]. It has been reported that early brain impairments and neurological deficits induced by intracerebral hemorrhage could be attenuated by regulating the activation of the NLRP3 inflammasome pathway via the stimulation of Nrf2 activity and the Nrf2-induced antioxidant system [30]. Additionally, endogenous Nrf2 stabilizing obviously dampened ROS-induced inflammasome activation in the kidneys of db/db mice. The decline of redox-sensitive transcription factor Nrf2 and the increase in its protein levels by minocycline inhibited inflammasome activation when abolished in diabetic Nrf2^−/−^ mice. Therapeutic approaches to diabetic nephropathy mainly target the inflammasome via Nrf2 activation or limit the proteasomal degradation of Nrf2 [31]. H_2_S plays a vital role in the regulation of the NLRP3 inflammasome and Nrf2 pathway activity. Liu et al. reported that H_2_S obviously attenuated the antioxidative capability, mitochondrial dysfunction, and acute liver injury induced by PQ via inhibiting ROS-induced NLRP3 inflammasome activation and promoting Nrf2-driven antioxidant enzyme pathway activation [17]. In our study, RIR induced the redox-sensitive transcription factor Nrf2 mRNA and protein activation, and NaHS treatment further activated Nrf2 mRNA and protein expression. To further discuss the effect of the Nrf2 pathway on NLRP3 and its downstream factors, Nrf2^−/−^ mice were used to perform the experiment. Nrf2 downstream factors HO-1, NQO1, and Trx were activated by RIR stimulation, and NaHS treatment further increased their expression in WT mice. Meanwhile, the effects of NaHS on HO-1, NQO1, and Trx were abolished in Nrf2^−/−^ mice. In addition, NaHS inhibited the NLRP3 inflammasome pathway induced by RIR in WT mice rather than Nrf2^−/−^ mice. These results support the hypothesis that NaHS inhibits NLRP3 pathway activation via the Nrf2 pathway in the lung tissue after RIR injury. We also investigated the effect of Nrf2 and NLRP3 on methandriol function, apoptosis, and lung injury after NaHS treatment. The results indicated that NaHS improved mitochondrial morphology, mitochondrial dysfunction, cell apoptosis, lung histology injury, lung edema and function, BALF cell counts, neutrophil counts, neutrophil elastase activity, and protein concentration in WT mice compared to in Nrf2^−/−^ mice. MCC950 treatment recovered the effect of NaHS and abolished the deterioration of the above indicators in the absence of Nrf2.

In summary, RIR induced remote lung tissue injury, mitochondrial dysfunction, and apoptosis and activated the Nrf2 and NLRP3 pathways. Exogenous H_2_S exerts protective effects on lung injury by regulating mitochondrial function and inflammation, which is involved in Nrf2 activation-mediated NLRP3 pathway inhibition. These findings will shed light on the role of H_2_S as a therapeutic agent for the protection of remote organs against RIR.

## Data Availability

The datasets used and/or analyzed during the current study are available from the corresponding author upon reasonable request.
